# Prognostic impacts and dynamic changes of cohesin complex gene mutations in de novo acute myeloid leukemia

**DOI:** 10.1038/s41408-017-0022-y

**Published:** 2017-12-29

**Authors:** Xavier Cheng-Hong Tsai, Hsin-An Hou, Jih-Luh Tang, Yuan-Yeh Kuo, Yu-Chiao Chiu, Chien-Chin Lin, Chieh-Yu Liu, Mei-Hsuan Tseng, Tzung-Yi Lin, Ming-Chih Liu, Chia-Wen Liu, Liang-In Lin, Ming Yao, Chi-Cheng Li, Shang-Yi Huang, Bor-Sheng Ko, Szu-Chun Hsu, Chien-Ting Lin, Shang-Ju Wu, Chien-Yuan Chen, Woei Tsay, Eric Y. Chuang, Wen-Chien Chou, Hwei-Fang Tien

**Affiliations:** 1 Division of Hematology, Department of Internal Medicine, National Taiwan University Hospital, Taipei, Taiwan; 2 Tai-Cheng Stem Cell Therapy Center, National Taiwan University, Taipei, Taiwan; 3 Genome and Systems Biology Degree Program, National Taiwan University, Taipei, Taiwan; 4 Graduate Institute of Oncology, National Taiwan University, Taipei, Taiwan; 5 Graduate Institute of Biomedical Electronics and Bioinformatics, National Taiwan University, Taipei, Taiwan; 6 Department of Laboratory Medicine, National Taiwan University Hospital, Taipei, Taiwan; 7 Graduate Institute of Clinical Medicine, National Taiwan University, Taipei, Taiwan; 8 Biostatistics Consulting Laboratory, School of Nursing and Center of General Education, National Taipei University of Nursing and Health Sciences, Taipei, Taiwan; 9 Department of Pathology, National Taiwan University Hospital, Taipei, Taiwan; 10 Department of Clinical Laboratory Sciences and Medical Biotechnology, College of Medicine, National Taiwan University, Taipei, Taiwan; 11 Bioinformatics and Biostatistics Core, Center of Genomic Medicine, National Taiwan University, Taipei, Taiwan

**Keywords:** Cancer genetics, Acute myeloid leukaemia

Cohesin complex is a multimeric protein complex, composed of four core subunits, including SMC1A, SMC3, RAD21, and either STAG1 or STAG2 proteins. They form a ring-shaped structure, and mediate sister chromatid cohesion and segregation during mitosis and meiosis. Recently, the cohesin gene mutations have been reported in myeloid neoplasms^1–7^, but studies regarding their clinical and prognostic relevance and dynamic changes in de novo acute myeloid leukemia (AML) patients are limited and the findings are controversial.

In this study, we aimed to investigate the clinical, biological, and prognostic implications of cohesin gene mutations in a large cohort of de novo AML patients. To evaluate the sequential changes of cohesin and co-occurring gene mutations, serial analyses of gene mutations by targeted next-generation sequencing (NGS) were performed in 386 samples from 116 patients during follow-ups. To the best of our knowledge, this is the first report to address the dynamic changes of cohesin gene mutations during the clinical course in de novo AML. We also investigated the pathophysiological pathways by mRNA expression profiling.

A total of 391 consecutive patients with newly diagnosed de novo non-M3 AML, consisting of 217 males and 174 females, were recruited. The coding sequences of cohesin complex genes were screened by Ion Torrent NGS (Thermo Fisher Scientific, MA, USA). All mutations were confirmed by Sanger sequencing. For non-synonymous missense mutations, we included only those reported to be pathogenic in literature, but not those predicted to be pathogenic solely by computational tools. Thirty-seven patients (9.5%) had cohesin gene mutations, most commonly in *RAD21* (15 of 391, 3.8%), followed by *STAG2* (12 of 390, 3.1%) and *SMC1A* (8 of 391, 2.0%). Except for one patient with concurrent mutations in *STAG2* and *RAD21*, the mutations in these component genes were mutually exclusive, suggesting a convergence of biological effects of these mutations (Fig. 1a). Mutations in *STAG2* and *RAD21* were mainly truncations or frameshift mutations (10/12 and 12/15, respectively), while those in *SMC1A* and *SMC3* were mostly missense mutations (7/8 and 2/2, respectively; Fig. 1b).

**Fig. 1:**
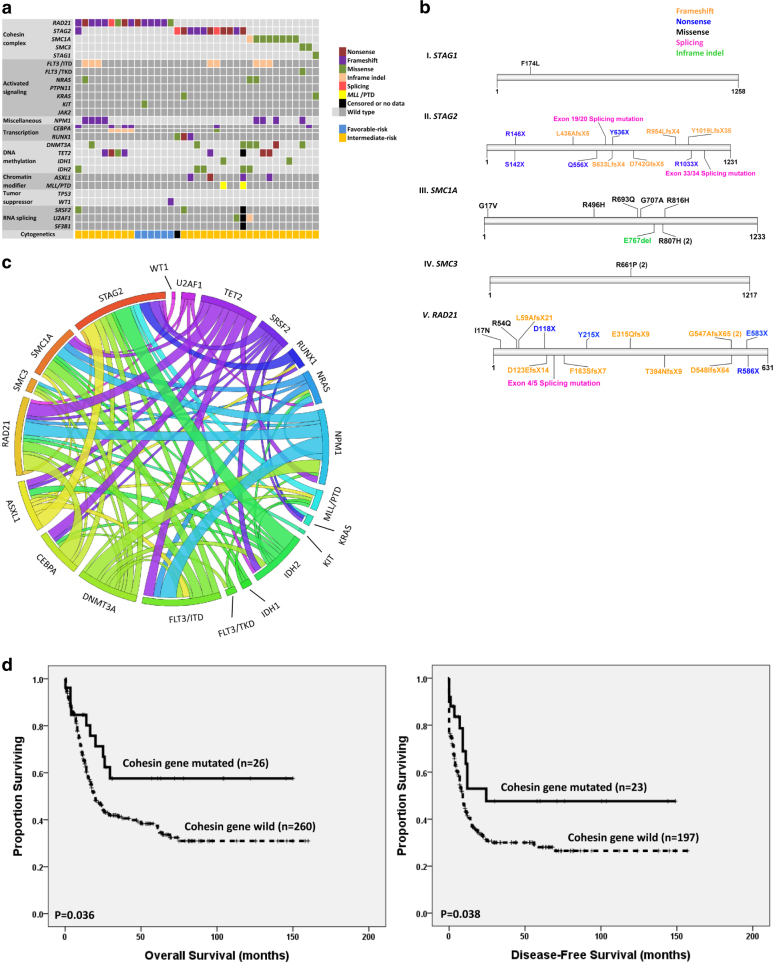

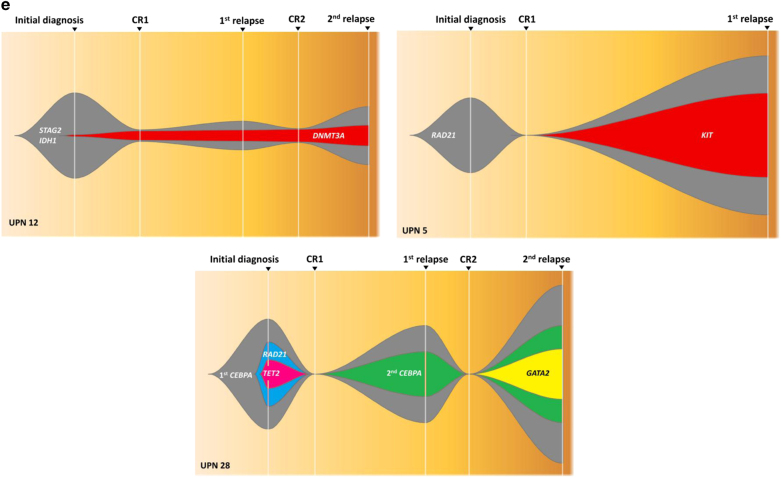
Cohesin complex gene mutations in de novo AML patients **a** The diagram of the associations in patients with cohesin gene mutations. The component gene mutations were almost mutually exclusive. The *MLL*/PTD was detected by polymerase chain reaction method. **b** The patterns and locations of cohesin gene mutations. **c** The frequencies and pairwise co-occurrence of genetic alterations in patients with cohesin gene mutations. **d** Kaplan–Meier survival curves for OS and DFS stratified by cohesin gene mutation status in the 286 de novo non-M3 adult AML patients who received standard intensive chemotherapy. **e** Graphical representations of clonal evolution of three cohesin gene-mutated patients. UPN 12 and UPN 5 had dominant clones with cohesin gene mutations, which retained at relapse. UPN 28 had a subclone with *RAD21* mutation at diagnosis, which disappeared during follow-up

Cohesin gene mutations were mutually exclusive with unfavorable-risk cytogenetics as well as complex chromosomal changes (*P* = 0.003 and *P* = 0.023, respectively, Supplementary Table S1), against that cohesin gene mutations lead to premature sister chromatid separation in AML. Therefore, cohesin gene mutations may take part in leukemogenesis by alternative mechanisms. Interestingly, six (16.7%) of the thirty-eight patients with t(8;21) had cohesin gene mutations, all in *RAD21*, while none of the patients with inv(16) had any cohesin mutation, compatible with previous reports^1,8^, indicating that concerted interaction of cohesin gene mutations with *RUNX1-RUX1T1* fusion plays a role in the leukemogenesis of some AML patients with t(8;21). In the 34 patients with acute promyelocytic leukemia and t(15;17) who were excluded from this study, none harbored a cohesin gene mutation (0% vs. 9.5%, *P* = 0.059, data not shown).

We screened mutations in 20 other genes, including *FLT3*, *NPM1*, *CEBPA, RUNX1*, *ASXL1*, *IDH1*, *IDH2*, *TET2*^9^, *DNMT3A*, *NRAS*, *KRAS*, *JAK2*, *KIT*, *PTPN11*, *SRSF2*, *U2AF1*, *SF3B1*^10^, *WT1*, *TP53*, and *MLL/*PTD^11^, to investigate the difference of the mutation profiles between cohesin gene-mutated and wild-type (WT) AML patients. Among the 37 patients with cohesin gene mutations, 30 (81.1%) patients had at least one other gene mutation simultaneously. The most common concurrent molecular events in cohesin gene-mutated cohort were *FLT3*/ITD (21.6%) and *NPM1* mutations (21.6%). None of the patients with cohesin gene mutations had *TP53* mutations. Compared with other cohesin gene mutations, *STAG2* mutations more frequently co-occurred with *RUNX1* mutations (27.3% vs. 0%, *P* = 0.023) and tended to co-occur with *ASXL1* mutations (25.0% vs. 4.0%, *P* = 0.084), but less frequently with *NPM1* mutations (0% vs. 32.0%, *P* = 0.036; Fig. 1a and Supplementary Table S2).

Until now, reports regarding prognostic relevance of cohesin gene mutations in AML are very limited. In this study, survival analyses were performed in the 286 (73.1%) patients who received standard chemotherapy, including 26 cohesin gene-mutated and 260 WT patients. The complete remission (CR), induction death, and relapse rate were similar between these two groups (Supplementary Table S3). With a median follow-up time of 53.0 months (range, 0.1–160), patients with cohesin gene mutations had significantly longer overall survival (OS) and disease-free survival (DFS) than those without the mutation (not reached vs. 20.0 ± 2.3 months, *P* = 0.036 and 24.5 ± 0.0 vs. 9.0 ± 0.8 months, *P* = 0.038, respectively, Fig. 1d). The detail of univariate analysis for OS and DFS was shown in Supplementary Table S4.

In multivariate Cox proportional hazards regression analysis for OS and DFS, cohesin gene mutations were independent favorable factors for both OS and DFS (Table 1). These results were in contrast to the report of Thol et al. in which cohesin gene mutations had no significant implication on OS and DFS (OS hazard ratio (HR) 0.96, *P* = 0.89 and RFS HR 0.62, *P* = 0.18, respectively)^5^. In that report, the incidence (5.9%) of cohesin gene mutations in AML was relatively low, compared with that in other reports (8.8–13.3%)^4,6,7^, and patients with secondary AML were included. Thota et al. reported that in patients with myelodysplastic syndrome (MDS) who survived more than 12 months, cohesin gene mutations were associated with a shorter survival (HR 2.1, *P* = 0.017). The prognostic implication of cohesin gene mutations in primary AML patients (*n* = 101) was not analyzed in that report^6^. The reasons why the cohesin gene mutations have opposite prognostic impact on AML and MDS remain unknown. Similar findings have been found in *SF3B1* mutation, which has a negative impact on de novo AML patients^2,10^, but a favorable impact on patients with MDS^12^.

**Table 1:** Multivariate analysis of the disease-free survival and overall survival

**Variables**	**Disease-free survival**				**Overall survival**			
	**RR**	**95% CI**		**P value**	**RR**	**95% CI**		**P value**
		**Lower**	**Upper**			**Lower**	**Upper**	
Total cohort (*n* = 286)								
Age^a^	1.916	1.401	2.621	<0.001	2.462	1.755	3.453	<0.001
WBC^b^	1.356	0.983	1.871	0.063	1.552	1.096	2.198	0.013
Karyotype^c^	1.553	0.982	2.456	0.060	1.936	1.219	3.076	0.005
*NPM1/FLT3*-ITD^d^	0.260	0.121	0.560	0.001	0.240	0.097	0.592	0.002
*CEBPA* ^double mutations e^	0.504	0.301	0.844	0.009	0.352	0.182	0.678	0.002
*RUNX1* ^e^	0.977	0.593	1.611	0.928	1.012	0.593	1.728	0.965
*ASXL1* ^e^	0.973	0.563	1.683	0.922	1.134	0.646	1.990	0.662
*IDH2* ^e^	0.845	0.518	1.377	0.498	0.490	0.260	0.924	0.028
Cohesin gene^e^	0.487	0.256	0.926	0.028	0.489	0.242	0.992	0.047
SF^e^	1.992	1.252	3.171	0.004	1.702	1.006	2.879	0.048
*TP53* ^e^	1.512	0.817	2.797	0.188	1.697	0.916	3.146	0.093

*RR* relative risk, *CI* confidence interval, *SF* splicing factor genes

^a^Age > 50 years relative to age ≤ 50 years (the reference)

^b^WBC > 50,000/μl vs. ≤50,000/μl

^c^Unfavorable cytogenetics vs. others

^d^*NPM1*^+^*/FLT3-*ITD^−^ vs. other subtypes

^e^Mutated vs. wild type

In order to evaluate the dynamic changes of cohesin and co-occurring gene mutations, we serially analyzed 386 samples from 116 patients, including 19 with and 97 without cohesin gene mutations at diagnosis (Table 2), for 54 gene mutations involved in myeloid malignancies by TruSight Myeloid Panel (Illumina, San Diego, CA, USA). HiSeq platform (Illumina) was used for sequencing with a median reading depth of 12,000×. Among the patients with cohesin gene mutations, 17 patients lost the original mutations at CR, while the mutations remained detectable at CR in UPN 1 and 12 (Fig. 1e), although with lower allele frequencies. The disease subsequently relapsed in these two patients with rising mutant allele burdens indicating the presence of minimal residual disease. Most other concurrent mutations in the 19 patients studied disappeared at CR but *DNMT3A* mutations were detectable in four patients (UPN 12, 16, 20, and 22), in whom *IDH1* (UPN 12), *U2AF1* (UPN 20 in CR2), or *NPM1* and *NRAS* mutations (UPN 22) also remained detectable at the same time. Two (UPN 28 and 37) of the eight cohesin-mutated patients who had paired samples at both diagnosis and relapse lost the original cohesin gene mutations (both in *RAD21*) during disease evolution. Graphical representations of clonal evolution in three representative patients were shown in Fig. 1e. Among the 97 patients without cohesin gene mutations at diagnosis, no one acquired the mutation at relapse, indicating that the mutations played little role in the progression of AML.

**Table 2:** Sequential studies in the AML patients with cohesin gene mutations at diagnosis^a^

**UPN** ^a^	**Interval** ^b^ **(months)**	**Disease status**	**Karyotype**	**Mutations**	
				**Cohesin**	**Others**
1		Diagnosis	49, XY, t(6;11)(q27;q23),+8,+9,+19	*STAG1* (F174L)	*KRAS*
	0.8	CR1	46, XY	*STAG1* (F174L)	—
	2.0	Relapse1	46, XY	*STAG1* (F174L)	—
5		Diagnosis	46, XY, t(8;21)(q22;q22)	*RAD21* (Y215Ter)	—
	0.9	CR1	46, XY	—	—
	12.0	Relapse1	46, XY, t(8;21)(q22;q22)	*RAD21* (Y215Ter)	*KIT*
6		Diagnosis	NM	*STAG2* (exon 19/20 splicing)	*BCOR*, *BCORL1*, *CSF3R*, *RUNX1*
	1.0	CR1	46, XY	—	—
9		Diagnosis	46, XY	*STAG2* (D742GfsTer5), *RAD21* (G547AfsTer65)	*IDH2*, *SRSF2*, *CEBPA*
	2.6	CR1	46, XY	—	—
11		Diagnosis	46, XY	*STAG2* (S633LfsTer4)	*BCOR*, *DNMT3A*, *IDH2*
	14.0	Relapse1	46, XY	*STAG2* (S633LfsTer4)	*BCOR*, *DNMT3A*, *IDH2*
	6.8	CR2	46, XY	—	—
12		Diagnosis	46, XY	*STAG2* (Q556Ter)	*IDH1*, *DNMT3A*
	1.1	CR1	ND	*STAG2* (Q556Ter)	*IDH1*, *DNMT3A*
	8.2	Relapse1	ND	*STAG2* (Q556Ter)	*IDH1*, *DNMT3A*
	4.4	CR2	ND	*STAG2* (Q556Ter)	*IDH1*, *DNMT3A*
	2.3	Relapse 2	ND	*STAG2* (Q556Ter)	*IDH1*, *DNMT3A*
16		Diagnosis	46, XY	*SMC3* (R661P)	*DNMT3A*, *FLT3*/TKD, *NPM1*
	1.3	CR1	46, XY	—	*DNMT3A*
18		Diagnosis	46, XX	*SMC1A* (R496H)	*FLT3*/ITD, *NPM1*, *TET2*
	1.0	CR1	ND	—	—
19		Diagnosis	46, XY	*SMC1A* (G707A)	—
	10.0	CR1	46, XY	—	—
20		Diagnosis	47, XY,+8	*SMC1A* (E767del)	*DNMT3A*, *IDH2*, *NRAS*, *U2AF1*
	1.7	CR1	47, XY	—	*DNMT3A*
	13.8	Relapse1	48, XY,+8,+15	*SMC1A* (E767del)	*DNMT3A*, *IDH2*, *NRAS*, *U2AF1*
	2.1	CR2	ND	—	*DNMT3A*, *U2AF1*
	8.1	Relapse2	48, XY,+X,+15	*SMC1A* (E767del)	*DNMT3A*, *IDH2*, *NRAS*, *U2AF1*, *RUNX1*, *CUX1*
22		Diagnosis	45, X,−Y	*SMC1A* (R693Q)	*DNMT3A*, *NPM1*, *NRAS*, *FLT3*/ITD
	0.9	CR1	NM	—	*DNMT3A*, *NPM1*, *NRAS*
	23.9	Relapse1	45, X,−Y	*SMC1A* (R693Q)	*DNMT3A*, *NPM1*, *FLT3*/ITD
	1.2	CR2	46, XY	—	—
23		Diagnosis	46, XY	*SMC1A* (G17V)	*IDH1*
	1.2	CR1	46, XY	—	—
24		Diagnosis	45, X,−Y	*SMC1A* (R816H)	*NPM1, DNMT3A*
	1.7	CR1	46, XY	—	—
27		Diagnosis	46, XY	*RAD21* (D118Ter)	*CEBPA*, *GATA2*, *TET2*
	1.0	CR1	ND	—	—
28		Diagnosis	46, XY	*RAD21* (I17Asn)	*CEBPA*^c^, *TET2*
	1.0	CR1	ND	—	—
	9.1	Relapse1	ND	—	*CEBPA* ^c^
	2.8	CR2	ND	—	—
	6.0	Relapse2	ND	—	*CEBPA* ^c^, *GATA2*
29		Diagnosis	46, XY	*RAD21* (exon 4/5 splicing)	*CEBPA*, *CSF3R*, *TET2*
	1.5	CR1	ND	—	—
32		Diagnosis	46, XX	*RAD21* (R54Q)	*PHF6*, *WT1*, *ETV6*
	84.7	CR1	46, XX	—	—
36		Diagnosis	46, XX, t(8;21)	*RAD21* (D548IfsTer64)	*PTPN11*, *KDM6A*
	5.0	CR1	46, XX	–	—
37		Diagnosis	46, XX, del(11)(q14q23)	*RAD21* (T394NfsTer9)	*CEBPA*
	1.0	CR1	46, XX	—	—
	5.0	Relapse1	46, XX	—	—

*UPN* unique patient number, *CR* complete remission, *ND* not done, *NM* no mitosis

^a^The data of serial studies in other 97 patients who did not have cohesin gene mutation at diagnosis were not shown in this table. None of them acquired a cohesin gene mutation at relapse

^b^Interval between the two successive studies

^c^UPN 28 had one *CEBPA* mutation at diagnosis (K313dup) but had two mutations at both relapse 1 and relapse 2 (K313dup and I68RfsTer39)

Furthermore, we applied Bradley–Terry model to evaluate the temporal order of gene mutations in cohesin-mutated patients (Supplementary Fig. S1). Only samples with statistically significant and recurrent gene–gene pairwise precedence were included in the analysis. The *STAG2* mutations occurred as an early event, while *RAD21* and *SMC1A* mutations occurred relatively late. In comparison of gene mutations between secondary and de novo AML, Lindsley et al. defined *STAG2* mutation as a secondary-type mutation because it was 95% specific for secondary AML^2^. However, other studies suggested that *STAG2* mutation might serve as an early event in leukemogenesis in AML^3,5,6,13,14^.

We further profiled genome-wide mRNA expression in 10 cohesin-mutated and 163 WT patients to explore the molecular mechanisms underlying cohesin gene mutations. One hundred and sixty-two differentially expressed genes were identified between the cohesin-mutated and WT AML (>1.5-fold change and *t*-test *P* < 0.05, Supplementary Fig. S2 and Supplementary Table S5). Ingenuity Pathway Analysis (IPA, Ingenuity Systems, Redwood City, CA, USA) and Gene Set Enrichment Analysis (GSEA) revealed that these genes were significantly associated with differentiation of blood cells, proliferation of blood cells, apoptosis, and cell death of blood cells (Supplementary Table S6 and Supplementary Fig. S3). Besides, a network constructed by IPA showed *ERK1/2* was a hub gene among the differentially expressed genes, implying involvement of this multifunctional kinase in cohesin gene mutation-driven signaling (Supplementary Fig. S4A). *ERK1/2* still played a central role in the networks constructed by the differentially expressed genes between *STAG2*-mutated and WT patients, and *RAD21*-mutated and WT patients (Supplemental Figs. S4B and S4C).

In conclusion, our study showed that cohesin gene mutations were recurrent in de novo AML and had favorable impacts on both OS and DFS. Further, cohesin gene mutations were strongly associated with the biological function related to proliferation and differentiation of blood cells. Sequential analyses showed cohesin gene mutations might be lost during disease evolution in de novo AML patients, but none of the patients without the mutation acquired a novel one during the clinical course. Further prospective studies with larger cohorts are warranted to confirm our findings.

## Electronic supplementary material

Supplemental Material

Supplemental Table S6
