# Supplementary material for: Prognostic impacts and dynamic changes of cohesin complex gene mutations in de novo acute myeloid leukemia
Source: Blood Cancer J. 2017 Dec 29;7(12):663. doi: 10.1038/s41408-017-0022-y (PMC5802563; doi:10.1038/s41408-017-0022-y)
Supplement: Supplementary file 1 — Supplemental Material [file 41408_2017_22_MOESM1_ESM.docx]

**Supplemental Material**

**Supplemental Method 1**

**Supplemental Table 7**

**Supplemental Figure 13**

**Supplemental method**

1. **Ion Torrent next generation sequencing for cohesin gene mutations detection at diagnosis**

We used customized Ion AmpliSeq panel (Thermo Fisher Scientific, MA, USA) to generate target amplicon libraries for cohesin complex genes. In short, 30 ng of DNA, extracted from bone marrow (BM) mononuclear cells, was amplified by polymerase chain reaction (PCR) using the premixed Ion AmpliSeq primer pools and Ion AmpliSeq HiFi master mix (Ion AmpliSeq kit version 2.0). The multiplexed amplicons were ligated to adapters from the Ion Xpress barcoded adapters according to the manufacturer’s instructions. After ligation, the amplicons underwent nick-translation and additional library amplification by PCR to complete the linkage between adapters and amplicons. An Agilent 2100 Bioanalyzer high-sensitivity DNA kit (Agilent, Santa Clara, CA) was used to visualize the size range and determine the library concentration.

Both individual libraries and multiplexed barcoded libraries were amplified by emulsion PCR on Ion Sphere particles (ISPs) at a 1:1 ratio of total library molecules to ISPs (Ion PGM Template OT2 200 kit; Thermo Fisher Scientific). Positive templated ISPs were biotinylated during the emulsion PCR process, so that samples with an optimal templated signal ratio were then enriched with Dynabeads MyOne streptavidin C1 beads (Thermo Fisher Scientific). Eight barcoded samples were multiplexed and sequenced on an Ion 318 chip. The median reading depth is 800x with Ion Torrent platform.

All the mutations detected by NGS were confirmed by Sanger sequencing. If the allele frequencies of certain mutations were too low for Sanger sequencing to detect, Taq polymerase-amplified cloning([1](#_ENREF_1)) was used for confirmation.

1. **Illumina next generation sequencing for serial studies**

Genomic DNA extracted from BM mononuclear cells was analyzed for 54 genes involved in myeloid malignancies using TruSight Myeloid Panel (Illumina, San Diego, CA, USA), which covered full coding sequence of 15 genes and exonic hot spots for other 39 genes. There were 568 amplicons (length range: 250 bp) and the cumulative target region size was 141 kb. Individual samples were indexed with a unique barcode and PCR amplified. Enriched pools of samples were sequenced on the HiSeq platform (Illumina). The median reading depth was 12000x.

1. **Variant calling and annotation**

Raw sequence data was mapped to human genome reference GRCh37/hg19 using BWA-MEM([2](#_ENREF_2)) version 0.7.9. SAMtools([3](#_ENREF_3)) version 0.1.18 was used to determine the coverage depth of each base. The variant calling was accomplished by CallSomaticVariants 3.6.2 (Somatic Variant Caller, Illumina) or Genome Analysis Tool Kit (GATK) HaplotypeCaller.([4](#_ENREF_4)) For detection of the large insertion of *FLT3* and *KMT2A*, Pindel([5](#_ENREF_5)) v0.2.5b8 were used specifically for chromosome 13 and 11, respectively, with default setting.

Variant calls were annotated by RefSeq([6](#_ENREF_6)) database and were described according to the recommendations of Human Genome Variation Society (HGVS). BM cells from 20 donors of hematopoietic stem cell transplantation were used as controls. Sequence alignment of selected variants was manually examined with Integrative Genomics Viewer (IGV).([7](#_ENREF_7)) Variants reported in 1000 Genomes project([8](#_ENREF_8)) or Exome Aggregation Consortium (ExAC)([9](#_ENREF_9)) were excluded.

The threshold of allele frequency and reading depth for identification was 5% and 100, respectively. For missense mutations, if the reading depths are more than 500, variants were further classified into pathogenic, benign, and undetermined significance according to COSMIC database version 67 (http://cancer.sanger.ac.uk),([10](#_ENREF_10)) dbSNP version 138,([11](#_ENREF_11)) ClinVar,([12](#_ENREF_12)) PolyPhen-2,([13](#_ENREF_13)) and SIFT.([14](#_ENREF_14)) If the reading depths of the missense mutations were between 100 and 500, only those which were reported previously to be pathogenic in literature would be taken into analysis. For truncation or frameshift mutations, the analysis algorithm was according to the previous literature.([15](#_ENREF_15)) Mutations unable to be confirmed were classified as undetermined significance, and would not be further analyzed. Only pathogenic gene mutations would be taken into analysis.

1. **Genetic mutation analysis other than cohesin gene complex**

Analyses of mutations in genes involving in activated signaling pathways, such as *FLT*,([16](#_ENREF_16)) *NRAS*,([17](#_ENREF_17)) *KRAS*,([17](#_ENREF_17)) *JAK2*,([17](#_ENREF_17)) *KIT*,([18](#_ENREF_18)) and *PTPN11*([18](#_ENREF_18)); transcription factors, such as *CEBPA*([19](#_ENREF_19)) and *RUNX1*([20](#_ENREF_20)); splicing factors, including *SRSF2*, *U2AF1*, and *SF3B1*;([21](#_ENREF_21)) epigenetic modifications, including *MLL/*PTD,([22](#_ENREF_22)) *ASXL1*,([23](#_ENREF_23)) *IDH1*,([24](#_ENREF_24)) *IDH2*,([25](#_ENREF_25)) *TET2*,*(*[*26*](#_ENREF_26)*)* and *DNMT3A*;*(*[*1*](#_ENREF_1)*)* as well as *NPM1*,([27](#_ENREF_27)) *WT1*,([28](#_ENREF_28)) and *TP53*,([29](#_ENREF_29)) were performed as previously described.

1. **Integrated gene expression analysis and pathway analysis**

For each sample, 1.5 μg complementary RNA was hybridized to HumanHT-12 v4 Expression BeadChip (Illumina) according to the manufacturer’s instructions. We used Illumina BeadArray Reader to detect intensities of bead fluorescence, and used GenomeStudio v2010.1 Software (Illumina) to transform the results to numeric values. The datasets were deposited into Gene Expression Omnibus database (accession number GSE68469 and GSE71014).

We performed pathway analysis on the microarray data as previously described([30](#_ENREF_30)) to interrogate the expression signatures underlying cohesin gene mutations. Fold-change test and two-sided Student’s *t*-test were used to identify differentially expressed genes between the two groups of patients with and without cohesin gene mutations. The knowledge-based software Ingenuity Pathway Analysis (IPA, Ingenuity Systems, Redwood City, CA, USA) was used to analyze the differentially expressed genes for associated biological functions. We used Gene Set Enrichment Analysis (GSEA) software v2.1.0([31](#_ENREF_31)) to further validated the biological functions of interest. GSEA tests whether a predefined gene set is enriched in the observed whole-genome gene expression profile. In contrast to the pathway analysis focusing on only differentially expressed genes, GSEA measures the global trend of changes in the expression profile and can uncover modest and subtle changes. The gene sets applied in the study were manually gathered from the IPA database. We used the 2000-time random permutation test among genes to assess the statistical significance of degree of enrichment.

1. **Statistical analysis**

The discrete variables of patients with and without specific molecular alteration were compared using the Fisher exact test. If the continuous data were not normally distributed, Mann-Whitney U tests were used to compare continuous variables and medians of distributions. To evaluate the impact of molecular alterations on clinical outcome, only the patients who received conventional standard chemotherapy were included in analyses. Overall survival (OS) was measured from the date of first diagnosis to the date of last follow-up or death from any cause, whereas relapse was defined as a reappearance of at least 5% leukemic blasts in BM aspiration smears or new extramedullary leukemia in patients with a previously documented CR.([32](#_ENREF_32)) Disease-free survival (DFS) was measured from the date of CR until relapse from CR or death from any cause, whichever occurred first.([33](#_ENREF_33)) Multivariate Cox proportional hazards regression analysis was used to investigate independent prognostic factors for OS and DFS. A P value <0.05 was considered statistically significant. All statistical analyses were performed with the SPSS 20 (SPSS Inc., Chicago, IL, USA) and Statsdirect (Cheshire, England, UK).

**Supplemental Table S1. Association of cohesin gene mutations with cytogenetic abnormalities***

| **Variables** | **Total** | **cohesin gene*-*mutated** | **cohesin gene WT** | **P value** |
| --- | --- | --- | --- | --- |
| **Karyotype†** |  |  |  |  |
| Favorable | 55 (14.7%) | 6 (16.7%) | 49 (14.5%) | 0.625 |
| Intermediate | 263 (70.1%) | 30 (83.3%) | 233 (68.7%) | 0.067 |
| Unfavorable | 57 (15.2%) | 0 (0%) | 57 (16.8%) | 0.003 |
| Normal | 185 (49.3%) | 21 (58.3%) | 164 (48.4%) | 0.295 |
| Simple | 147 (39.2%) | 15 (41.7%) | 132 (38.9%) | 0.858 |
| Complex | 43 (11.5%) | 0 (0%) | 43 (12.7%) | 0.023 |
| t(8;21) | 38 (10.1%) | 6 (16.7%) | 32 (9.4%) | 0.238 |
| inv(16) | 17 (4.5%) | 0 (0%) | 17 (5.0%) | 0.390 |
| t(11q23) | 13 (3.5%) | 2 (5.6%) | 11 (3.2%) | 0.360 |
| t(7;11) | 9 (2.4%) | 1(2.8%) | 8 (2.4%) | 0.601 |
| -5/5q-‡ | 17 (4.5%) | 0 (0%) | 17 (5.0%) | 0.390 |
| -7/7q-‡ | 22 (5.9%) | 0 (0%) | 22 (6.5%) | 0.250 |
| +8‡ | 19 (5.1%) | 2 (5.6%) | 17 (5.0%) | 0.702 |
| +11‡ | 3 (0.8%) | 0 (0%) | 3 (0.9%) | >0.999 |
| +13‡ | 1 (0.3%) | 0 (0%) | 1 (0.3%) | >0.999 |
| +21‡ | 8 (2.1%) | 1 (2.8%) | 7 (2.1%) | 0.558 |

Abbreviations: WT, wild type.

* Cytogenetic data were available in 375 patients, including 36 with mutated cohesin genes and 339 with wild type cohesin genes at diagnosis.

^†^Favorable, t(8;21) and inv (16) ; unfavorable, -7, del(7q), -5, del(5q), 3q abnormality, and complex abnormalities; Intermediate, normal karyotype and other abnormalities.

^‡^Only including simple chromosomal abnormalities with 2 or less changes, but not those with complex abnormalities with 3 or more aberrations.

**Supplemental Table S2. Comparison of concurrent alterations of other genes between AML patients with and without cohesin gene mutations**

| **Variables** |  | | **No. of patients with alteration (%)** | | | **P value** |
| --- | --- | --- | --- | --- | --- | --- |
|  | **Total patients examined** | **Whole cohort** | | **cohesin gene*-*mutated**  **(n=37, 9.5%)** | **cohesin gene WT**  **(n=354, 90.5%)** |  |
| *FLT3/*ITD | 391 | 86 (22.0%) | | 8/37 (21.6%) | 78/354 (22.0%) | >0.999 |
| *FLT3/*TKD | 391 | 24 (6.1%) | | 1/37 (2.7%) | 23/354 (6.5%) | 0.715 |
| *NRAS* | 391 | 48 (12.3%) | | 3/37 (8.1%) | 45/354 (12.7%) | 0.599 |
| *KRAS* | 391 | 14 (3.6%) | | 1/37 (2.7%) | 13/354 (3.7%) | >0.999 |
| *PTPN11* | 391 | 18 (4.6%) | | 0/37 (0%) | 18/354 (5.1%) | 0.397 |
| *KIT* | 391 | 13 (3.3%) | | 1/37 (2.7%) | 12/354 (3.4%) | >0.999 |
| *JAK2* | 391 | 2 (0.5%) | | 0/37 (0%) | 2/354 (0.6%) | >0.999 |
| *WTI* | 391 | 25 (6.4%) | | 1/37 (2.7%) | 24/354 (6.8%) | 0.493 |
| *NPM1* | 391 | 84 (21.5%) | | 8/37 (21.6%) | 76/354 (21.5%) | >0.999 |
| *CEBPA* | 391 | 58 (14.8%) | | 7/37 (18.9%) | 51/354 (14.4%) | 0.467 |
| *RUNX1* | 391 | 53 (13.6%) | | 3/37 (8.1%) | 50/354 (14.1%) | 0.449 |
| *MLL/*PTD | 391 | 25 (6.4%) | | 2/37 (5.4%) | 23/354 (6.5%) | >0.999 |
| *ASXL1* | 391 | 46 (11.8%) | | 4/37 (10.8%) | 42/354 (11.9%) | >0.999 |
| *IDH1* | 391 | 26 (6.6%) | | 2/37 (5.4%) | 24/354 (6.8%) | >0.999 |
| *IDH2* | 391 | 45 (11.5%) | | 5/37 (13.5%) | 40/354 (11.3%) | 0.597 |
| *TET2* | 391 | 47 (12.1%) | | 7/37 (18.9%) | 40/354 (11.3%) | 0.175 |
| *DNMT3A* | 391 | 65 (16.6%) | | 7/37 (18.9%) | 58/354 (16.4%) | 0.647 |
| *TP53* | 391 | 32 (8.2%) | | 0/37 (0%) | 32/354 (9.0%) | 0.058 |
| SF | 385 | 46 (11.9%) | | 4/36 (11.1%) | 42/349 (12.0%) | >0.999 |

Abbreviations: WT, wild type; SF, splicing factor genes, including *SRSF2*, *U2AF1* and *SF3B1*.

**Supplemental table S3. Clinical features between AML patients with and without cohesin gene mutation**

| **Variables** | **Total**  **(n=391)** | **cohesin gene*-*mutated**  **(n=37, 9.5%)** | | **cohesin gene WT**  **(n=354, 90.5%)** | **P value** |
| --- | --- | --- | --- | --- | --- |
| **Sex**^†^ |  |  | |  |  |
| Male | 217 | 23 (62.2%) | | 194 (54.8%) | 0.487 |
| Female | 174 | 14 (37.8%) | | 160 (45.2%) |  |
| **Age (year)**^‡^ | 51.7 (18-90) | 55.0 (19-80) | | 51.0 (18-90) | 0.608 |
| **Lab data**^‡^ |  |  | |  |  |
| WBC (/μL) | 21810.0 (120.0-627800.0) | | 12570.0 (310.0-417500.0) | 22320.0 (120.0-627800.0) | 0.231 |
| Hb (g/dL) | 7.9 (3.0-16.0) | 8.2 (3.0-14.0) | | 7.9 (3.0-16.0) | 0.955 |
| Platelet (×1,000 /μL) | 44.0 (3.0-712.0) | 36.0 (6.0-314.0) | | 45.0 (3.0-712.0) | 0.131 |
| Blast (/μL) | 9802.0 (0-456724.5) | 3859.2 (54.4-369070.0) | | 10131.4 (0-456724.5) | 0.091 |
| LDH (U/L) | 860.0 (206.0-13130.0) | 773.0 (250.0-5970.0) | | 888.0 (206.0-13130.0) | 0.268 |
| **FAB**^†^ |  |  | |  |  |
| M0 | 10 | 1 (2.7%) | | 9 (2.5%) | >0.999 |
| M1 | 95 | 4 (10.8%) | | 91 (25.7%) | 0.045 |
| M2 | 149 | 17 (45.9%) | | 132 (37.3%) | 0.374 |
| M4 | 105 | 11 (29.7%) | | 94 (26.6%) | 0.698 |
| M5 | 21 | 2 (5.4%) | | 19 (5.4%) | >0.999 |
| M6 | 11 | 2 (5.4%) | | 9 (2.5%) | 0.279 |
| **Induction response*** |  |  | |  |  |
| CR | 220 | 23 (88.5%) | | 197 (75.8%) | 0.490 |
| PR/Refractory | 46 | 2 (7.7%) | | 44 (16.9%) | 0.287 |
| Induction death | 20 | 1 (3.8%) | | 19 (7.3%) | 0.708 |
| **Relapse** | 116 | 9 (39.1%) | | 107 (54.3%) | 0.190 |

Abbreviations: FAB, French-American-British classification; CR, complete remission; PR, partial remission; WT: wild type

^†^number of patients (%)

^‡^median (range)

*Only the 286 patients who received conventional chemotherapy, including 26 cohesin-mutated patients

and 260 cohesin wild type patients were included for analysis.

**Supplemental table S4. Univariate analysis on the overall survival and disease-free survival**

| **Variable^‡^** | **Disease-free Survival** | | | **Overall Survival** | | | | |
| --- | --- | --- | --- | --- | --- | --- | --- | --- |
|  | Months^#^ | P value | | | Months^#^ | P value | |  |
| **Age** |  | | <0.001 |  | | | <0.001 | |
| >50 years | 7.0±1.9 | |  | 13.0±1.8 | | |  | |
| ≤50 | 12.0±3.7 | |  | 43.0±14.4 | | |  | |
| **WBC at diagnosis** |  | | 0.040 |  | | | 0.021 | |
| > 50,000/μ | 7.5±2.3 | |  | 17.0±1.7 | | |  | |
| ≤ 50,000/μL | 11.0±1.7 | |  | 26.0±10.4 | | |  | |
| **Cytogenetic risk** |  | | <0.001 |  | | | <0.001 | |
| Unfavorable | 2.5±2.8 | |  | 9.5±1.1 | | |  | |
| Others | 10.0±1.0 | |  | 25.0±8.1 | | |  | |
| ***NPM1^+^/ FLT3-*ITD*^-^*** |  | | <0.001 |  | | | 0.002 | |
| Yes | NR | |  | NR | | |  | |
| Others | 9.0±0.8 | |  | 20.0±2.3 | | |  | |
| ***CEBPA*^double mutations^** |  | | 0.015 |  | | | 0.002 | |
| Yes | 59.0 | |  | NR | | |  | |
| Others | 9.0±0.8 | |  | 20.0±2.2 | | |  | |
| ***RUNX1*** |  | | 0.184 |  | | | 0.046 | |
| Mutated | 6.0±4.2 | |  | 12.3±3.4 | | |  | |
| Wild | 9.5±1.0 | |  | 25.0±3.2 | | |  | |
| ***WT1*** |  | | 0.027 |  | | | 0.167 | |
| Mutated | 5.0±2.0 | |  | 14.5±2.3 | | |  | |
| Wild | 10.0±1.5 | |  | 23.5±3.6 | | |  | |
| ***IDH2*** |  | | 0.664 |  | | | 0.035 | |
| Mutated | 12.0±3.2 | |  | NR | | |  | |
| Wild | 9.0±1.0 | |  | 20.0±2.2 | | |  | |
| ***ASXL1*** |  | | 0.454 |  | | | 0.031 | |
| Mutated | 8.0±3.5 | |  | 14.0±2.5 | | |  | |
| Wild | 9.0±1.2 | |  | 25.0±4.9 | | |  | |
| ***DNMT3A*** |  | | 0.174 |  | | | 0.325 | |
| Mutated | 4.0±2.3 | |  | 13.5±3.7 | | |  | |
| Wild | 9.5±0.9 | |  | 23.5±3.1 | | |  | |
| ***IDH1*** |  | | 0.712 |  | | | 0.930 | |
| Mutated | 15.0±2.7 | |  | 20.0±1.5 | | |  | |
| Wild | 9.0±1.1 | |  | 23.0±2.7 | | |  | |
| ***TET2*** |  | | 0.573 |  | | | 0.087 | |
| Mutated | 9.0±1.1 | |  | 14.0±2.7 | | |  | |
| Wild | 9.0±1.3 | |  | 23.0±3.5 | | |  | |
| ***PTPN11*** |  | | 0.354 |  | | | 0.288 | |
| Mutated | 0.0 | |  | 6.5±8.9 | | |  | |
| Wild | 9.0±1.0 | |  | 23.0±3.0 | | |  | |
| ***RAS***^Ψ^ |  | | 0.323 |  | | | 0.959 | |
| Mutated | 9.0±1.6 | |  | 25.0±4.6 | | |  | |
| Wild | 9.0±1.3 | |  | 22.0±3.9 | | |  | |
| ***KIT*** |  | | 0.688 |  | | | 0.896 | |
| Mutated | 9.0±3.2 | |  | 15.0±7.3 | | |  | |
| Wild | 9.0±1.0 | |  | 22.0±3.1 | | |  | |
| ***MLL-*PTD** |  | | 0.334 |  | | | 0.467 | |
| Mutated | 7.5±3.3 | |  | 16.5±3.1 | | |  | |
| Wild | 9.5±1.0 | |  | 23.5±3.3 | | |  | |
| ***TP53*** |  | | 0.001 |  | | | <0.001 | |
| Mutated | 0.0 | |  | 7.0±3.0 | | |  | |
| Wild | 10.0±0.9 | |  | 25.0±4.8 | | |  | |
| **Cohesin gene** |  | | 0.038 |  | | | 0.036 | |
| Mutated | 24.5 | |  | NR | | |  | |
| Wild | 9.0±0.8 | |  | 20.0±2.3 | | |  | |
| **SF gene** |  | | <0.001 |  | | | 0.001 | |
| Mutated | 0.0 | |  | 8.0±4.1 | | |  | |
| Wild | 10.0±1.2 | |  | 25.0±5.7 | | |  | |

Abbreviation: NR, not reached; SF: splicing factor genes.

^#^Months: median±standard deviation

*Statistically significant (P < 0.05)

^Ψ^Including *NRAS* and *KRAS*

**Supplemental Table S5.**

**The unique gene list with differential expression between cohesin gene-mutated and wild groups**

| **Gene Name** | ***P* value** | **Gene Name** | ***P* value** | **Gene Name** | ***P* value** | **Gene Name** | ***P* value** | **Gene Name** | ***P* value** | **Gene Name** | ***P* value** | **Gene Name** | ***P* value** |
| --- | --- | --- | --- | --- | --- | --- | --- | --- | --- | --- | --- | --- | --- |
| *TUBB6* | 0.0387 | *IFI27L2* | 0.018184 | *PADI4* | 0.0133 | *CYP27A1* | 6.95E-05 | *FTHL2* | 0.028627 | *LOC729009* | 0.040552 | *ALDH4A1* | 0.039245 |
| *F13A1* | 0.012243 | *PVRL2* | 8.67E-05 | *LAT2* | 0.019529 | *WIT1* | 0.020339 | *IRAK2* | 0.015999 | *KENAE* | 0.013632 | *FTHL12* | 0.024111 |
| *PALM* | 0.004126 | *ZNF185* | 0.036275 | *S100Z* | 0.007132 | *GHRL* | 0.027087 | *TCEA3* | 0.019917 | *HTATIP2* | 0.001321 | *RASL10A* | 0.004387 |
| *TPSAB1* | 0.047532 | *PRDM8* | 0.000283 | *FKBP9L* | 0.009826 | *NINJ2* | 0.018037 | *RELL1* | 0.014374 | *LOC339192* | 0.010105 | *MIR21* | 0.000451 |
| *MIR1974* | 0.046518 | *RHOC* | 0.02511 | *KLF11* | 0.044484 | *SULF2* | 0.02649 | *FTHL8* | 0.013233 | *LOC100131289* | 0.021863 | *LOC728975* | 0.031212 |
| *LDLR* | 0.002342 | *SULF2* | 0.006699 | *BAIAP2* | 0.015781 | *ITGB5* | 0.005775 | *FYN* | 0.005359 | *CKS2* | 0.045581 | *LOC727908* | 0.015493 |
| *KLF11* | 0.029201 | *FNBP1L* | 0.016356 | *H3F3B* | 0.024339 | *CYORF15B* | 0.049726 | *FTHL11* | 0.005353 | *ESAM* | 0.007982 | *KIAA1715* | 0.045004 |
| *LGALS3BP* | 0.008569 | *ERAP2* | 0.000199 | *FTH1* | 0.001832 | *NCALD* | 0.021468 | *METRNL* | 0.038012 | *FRMD3* | 0.005519 | *TNFRSF21* | 0.044269 |
| *TPSB2* | 0.035285 | *PRIC285* | 0.014025 | *SMAGP* | 0.007318 | *ACVR1* | 0.0454 | *PVRL2* | 0.000204 | *GAB2* | 0.037814 | *C8ORF73* | 0.045256 |
| *HIST2H2BE* | 0.03407 | *CCDC109B* | 0.03564 | *ZNF521* | 0.017266 | *LOC652751* | 0.009611 | *FUT7* | 0.00319 | *DDAH2* | 0.01418 | *LY6E* | 0.047166 |
| *UGT3A2* | 0.037387 | *DSE* | 0.022911 | *ALDH4A1* | 0.018869 | *COL23A1* | 0.007758 | *ANGPTL6* | 0.001814 | *GNA12* | 0.003208 | *RN5S9* | 0.019666 |
| *EMP1* | 0.002629 | *CD9* | 0.037284 | *CPD* | 0.007228 | *LOC644936* | 0.034141 | *C20ORF117* | 0.003064 | *P2RY2* | 0.000931 | *HEYL* | 0.017128 |
| *ACSF3* | 0.02125 | *SQLE* | 0.000162 | *ERN1* | 0.002947 | *ZFHX3* | 0.002332 | *MTHFS* | 0.001114 | *SLC35F2* | 0.020482 | *DAB2* | 0.019893 |
| *LOC338758* | 0.029942 | *RASD1* | 0.027835 | *ATN1* | 0.0028 | *ALDH4A1* | 0.032686 | *BTG1* | 0.040066 | *DDEF2* | 0.04547 | *AIG1* | 0.049157 |
| *HBEGF* | 0.001296 | *FLNB* | 0.001995 | *FTHL3* | 0.012759 | *FAM101B* | 0.032876 | *BRI3BP* | 0.022159 | *HS.572219* | 0.012774 | *CA5B* | 0.021886 |
| *KLHDC8B* | 0.012067 | *PALM* | 0.000266 | *ITGB5* | 0.01623 | *FTHL11* | 0.012625 | *TSPAN32* | 3.58E-05 | *HS.403972* | 0.001066 | *DAB2* | 0.013469 |
| *SGK* | 0.042699 | *AEBP1* | 0.008071 | *FTHL12* | 0.009643 | *SULF2* | 0.01401 | *SRC* | 0.018923 | *CKS2* | 0.049568 | *DNAJB9* | 0.023207 |
| *HOMER3* | 0.018207 | *C20ORF201* | 0.037756 | *FTHL16* | 0.02517 | *ALDH4A1* | 0.010633 | *HERPUD1* | 0.043682 | *SLC24A3* | 0.00045 | *ALDH4A1* | 0.039245 |
| *TPSAB1* | 0.02415 | *LGALS3* | 0.019973 | *EGR2* | 0.005264 | *EPHB4* | 0.00781 | *ETS1* | 0.020028 | *RHOU* | 0.025702 | *FTHL12* | 0.024111 |
| *BIK* | 0.021832 | *MCL1* | 0.008132 | *SPRY2* | 0.021789 | *C15ORF52* | 0.012564 | *ZFP90* | 0.001207 | *SRPK2* | 0.036842 |  |  |
| *HS.576106* | 0.024153 | *TGFBI* | 0.00362 | *SV2B* | 0.015377 | *RHBDF1* | 0.024695 | *GRAP* | 0.003343 | *CCRK* | 0.018037 |  |  |
| *TNFAIP3* | 0.038616 | *PIM3* | 0.040334 | *MCL1* | 0.004584 | *MTSS1* | 0.00268 | *MTHFD2* | 0.04681 | *LSMD1* | 0.045974 |  |  |
| *DACH1* | 0.042907 | *CHST13* | 0.044313 | *GNA12* | 0.005854 | *SH2B3* | 0.01982 | *NUDT1* | 0.000658 | *GRASP* | 0.019591 |  |  |
| *ZNF385C* | 0.028502 | *RHOC* | 0.033582 | *IFI27L2* | 0.017155 | *LOC651894* | 0.03084 | *ATP2A2* | 0.016909 | *FAM45A* | 0.034705 |  |  |

**Supplemental Figure S1. The relative temporal order of gene mutations in recurrent pairwise precedence in cohesin-mutated patients.**


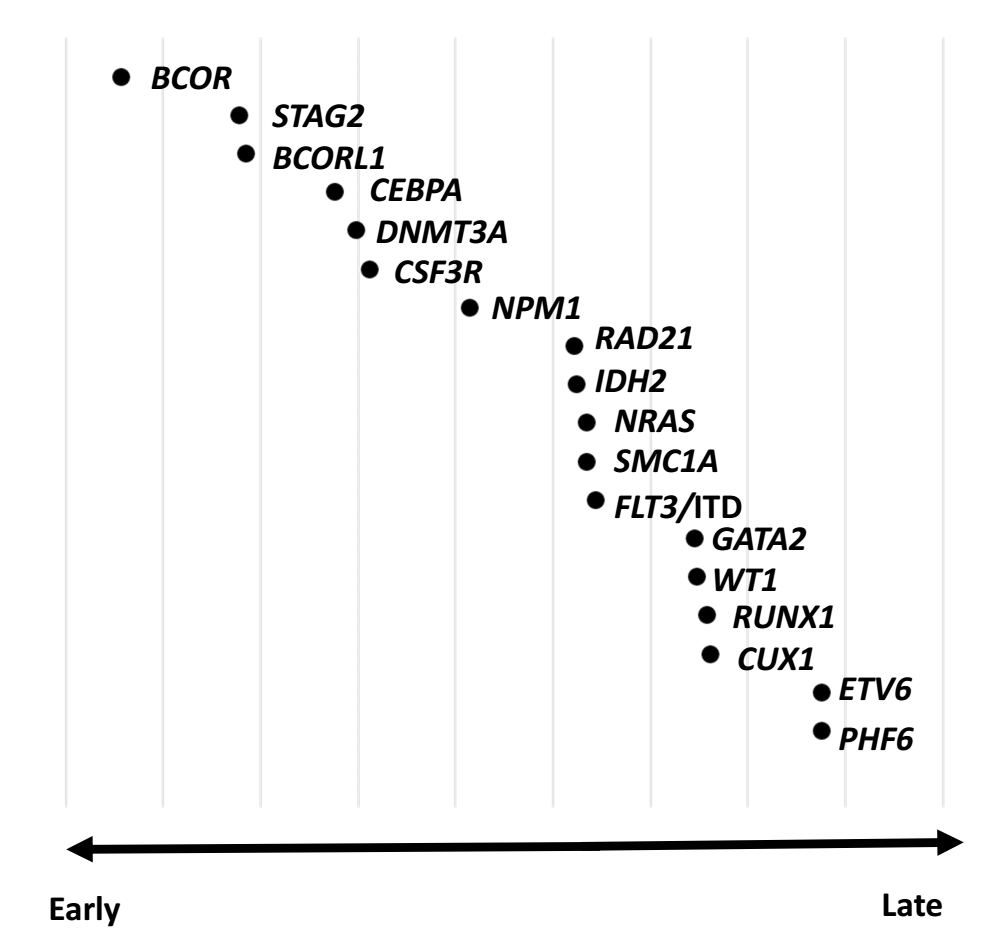
**Supplemental Figure S2. The heatmap of genes differential expressed between cohesin gene-mutated and WT patients.**


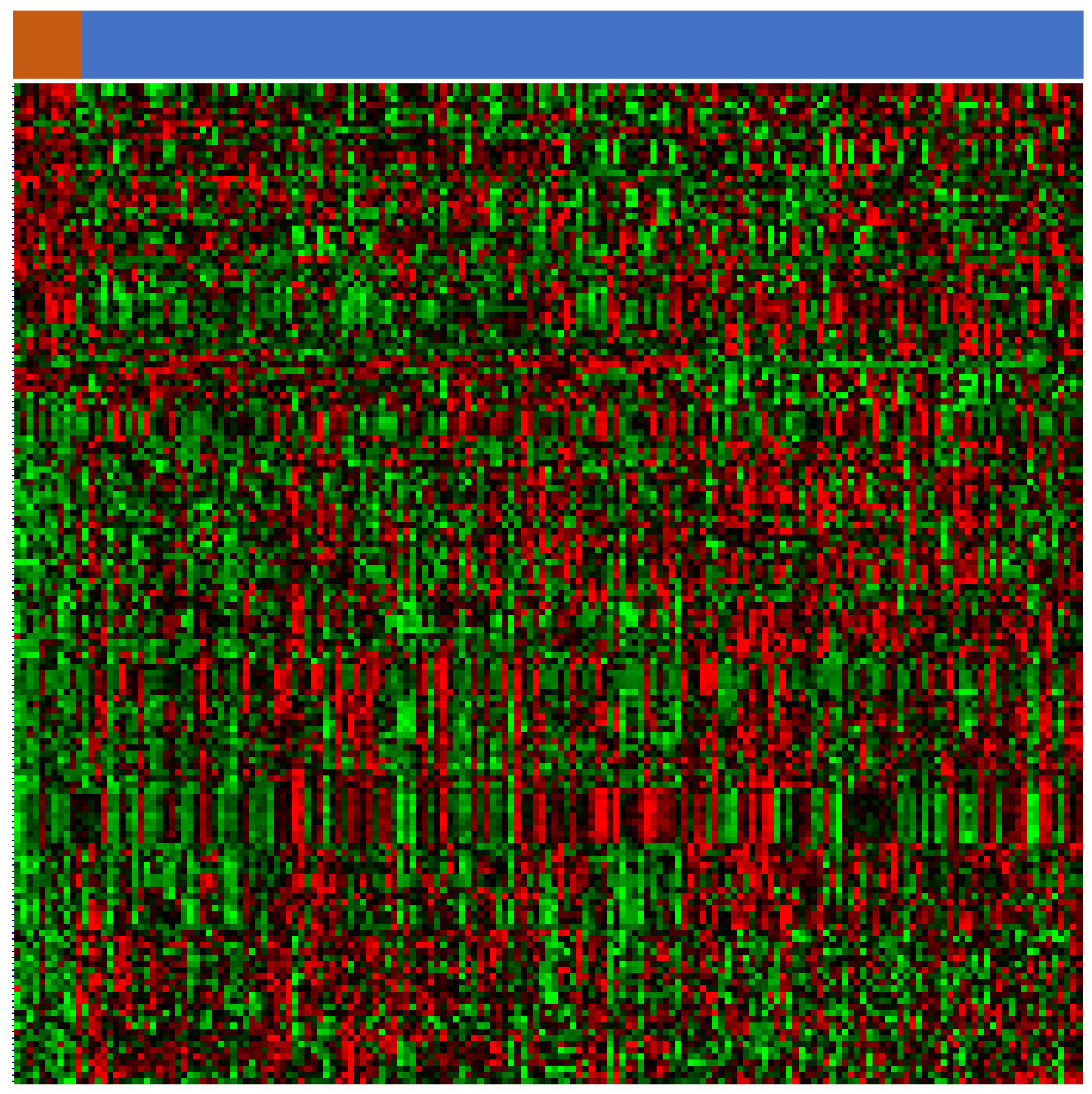


FUT7

TSPAN32

LAT2

LAT2

C15ORF52

GRAP

SRPK2

CCRK

HEYL

ERAP2

KENAE

KIAA1715

ZNF185

S100Z

ZFP90

SV2B

LGALS3BP

ANGPTL6

LOC100131289

ACSF3

CYORF15B

ZNF385C

GHRL

BRI3BP

C20ORF201

NUDT1

LSMD1

LOC651894

CKS2

CKS2

MIR1974

RN5S9

IFI27L2

IFI27L2

MTHFS

ALDH4A1

ALDH4A1

ALDH4A1

ALDH4A1

C8ORF73

COL23A1

FAM45A

UGT3A2

AIG1

LOC339192

CHST13

DDAH2

RASD1

KLHDC8B

RHBDF1

GNA12

GNA12

ITGB5

ITGB5

SRC

HS.403972

HOMER3

FAM101B

FKBP9L

PVRL2

PVRL2

BIK

WIT1

HS.572219

BAIAP2

ATN1

PRDM8

AEBP1

EPHB4

PALM

PALM

SLC24A3

DACH1

ZNF521

ETS1

HIST2H2BE

H3F3B

CPD

RELL1

MCL1

GAB2

FNBP1L

DSE

SPRY2

SLC35F2

MTHFD2

MIR21

CD9

LDLR

RHOC

RHOC

TUBB6

RASL10A

C20ORF117

ATP2A2

SQLE

EMP1

DDEF2

F13A1

ACVR1

CA5B

TGFBI

SULF2

SULF2

SULF2

SH2B3

HTATIP2

LOC644936

LOC727908

TPSAB1

TPSAB1

TPSB2

LOC652751

MTSS1

SMAGP

ESAM

LY6E

FLNB

TNFRSF21

HS.576106

NCALD

LOC728975

DAB2

DAB2

PADI4

NINJ2

FRMD3

P2RY2

PRIC285

KLF11

KLF11

EGR2

SGK

TNFAIP3

FTH1

MCL1

BTG1

HERPUD1

CCDC109B

FYN

CYP27A1

LGALS3

RHOU

LOC338758

METRNL

FTHL16

FTHL12

FTHL2

FTHL11

FTHL3

FTHL11

LOC729009

FTHL8

FTHL12

HBEGF

ZFHX3

TCEA3

ERN1

IRAK2

PIM3

GRASP

DNAJB9


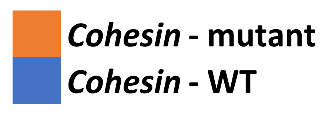
**Supplemental Figure S3. GSEA plots on genes associated with (a) differentiation of blood cells (b) proliferation of blood cells (C) apoptosis (D) cell death of blood cells.**

**(A)**


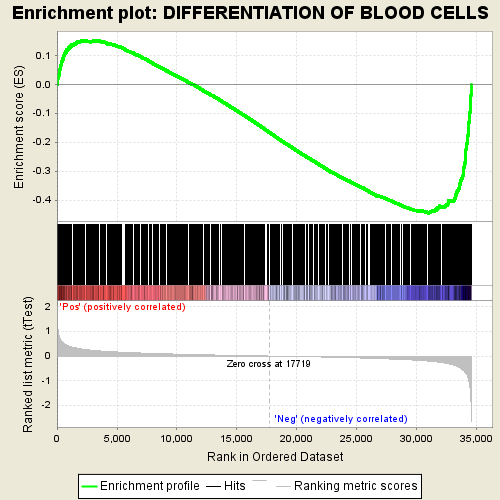


**NES= -1.94**

**P<0.001**

**(B)**


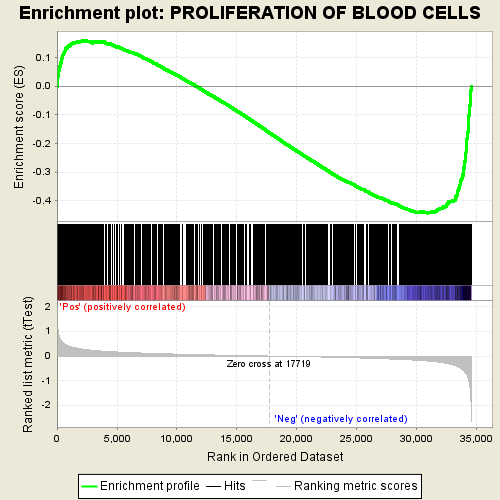


**P<0.001**

**NES= -1.93**

**(C)**


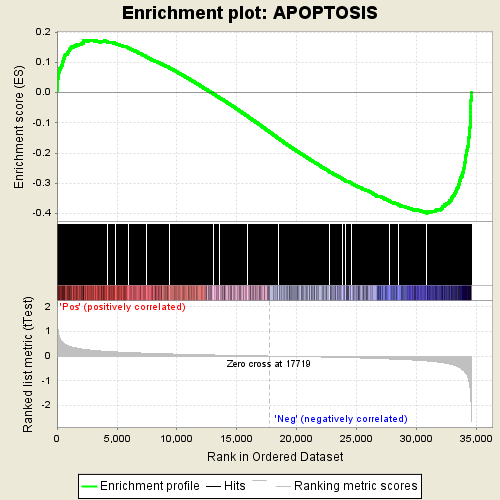


**(D)**

**P<0.001**

**NES= -1.77**


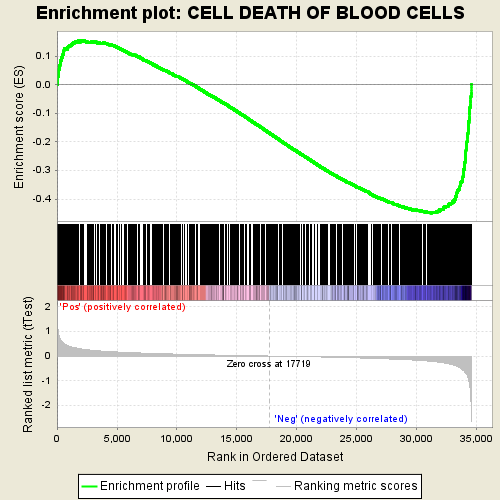


**NES= -1.94**

**Supplemental Figure S4. Mechanistic networks of (A) cohesin gene mutations as a whole, (B) *STAG2* mutations, and (C) *RAD21* mutations generated by IPA.**

**P<0.001**

**(A)**

**
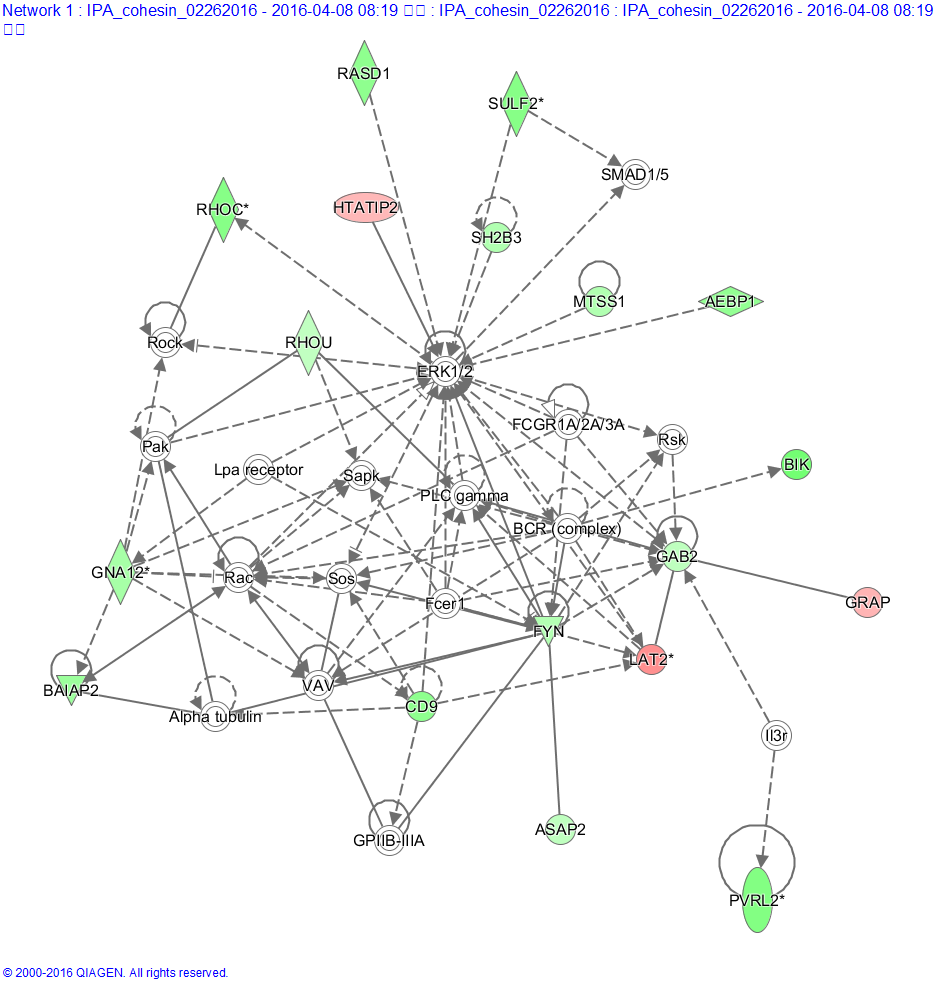
**


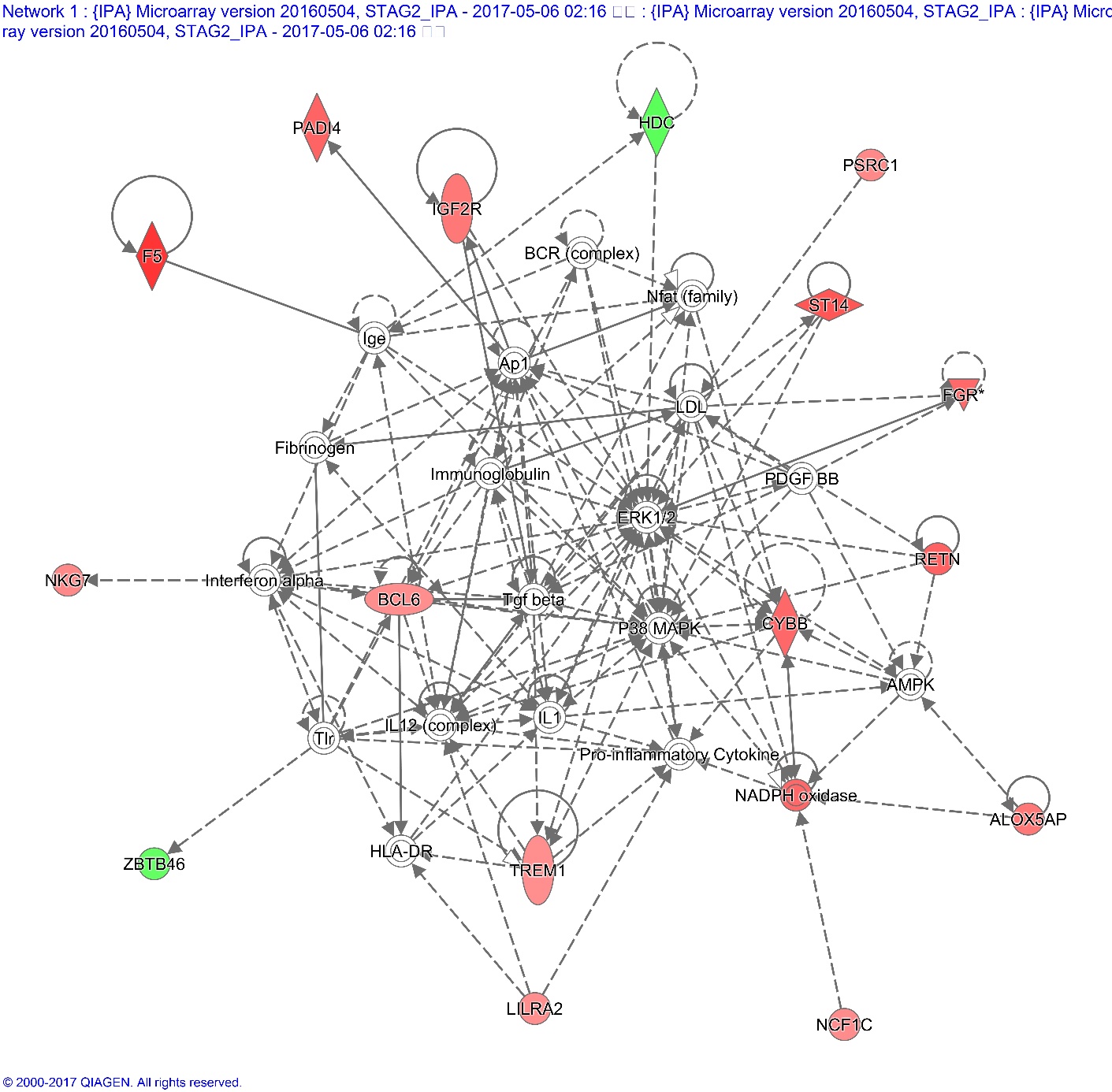
**(B)**

**(C)**


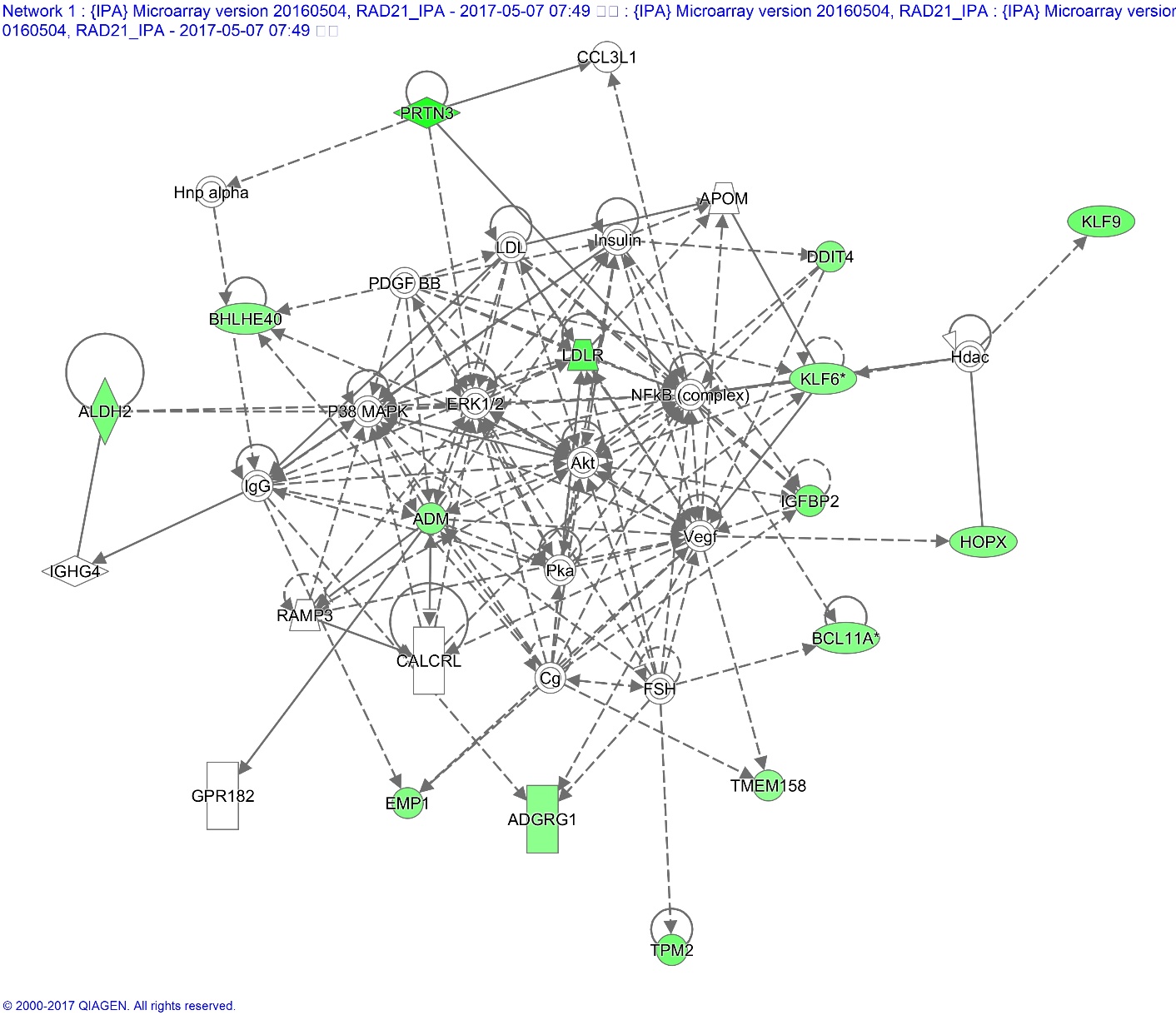


1. Hou HA, Kuo YY, Liu CY, Chou WC, Lee MC, Chen CY, et al. DNMT3A mutations in acute myeloid leukemia: stability during disease evolution and clinical implications. Blood. 2012;119(2):559-68.

2. Li H. Toward better understanding of artifacts in variant calling from high-coverage samples. Bioinformatics (Oxford, England). 2014;30(20):2843-51.

3. Li H, Handsaker B, Wysoker A, Fennell T, Ruan J, Homer N, et al. The Sequence Alignment/Map format and SAMtools. Bioinformatics (Oxford, England). 2009;25(16):2078-9.

4. DePristo MA, Banks E, Poplin R, Garimella KV, Maguire JR, Hartl C, et al. A framework for variation discovery and genotyping using next-generation DNA sequencing data. Nat Genet. 2011;43(5):491-8.

5. Basara N, Schulze A, Wedding U, Mohren M, Gerhardt A, Junghanss C, et al. Early related or unrelated haematopoietic cell transplantation results in higher overall survival and leukaemia-free survival compared with conventional chemotherapy in high-risk acute myeloid leukaemia patients in first complete remission. Leukemia. 2009;23(4):635-40.

6. O'Leary NA, Wright MW, Brister JR, Ciufo S, Haddad D, McVeigh R, et al. Reference sequence (RefSeq) database at NCBI: current status, taxonomic expansion, and functional annotation. Nucleic Acids Res. 2016;44(D1):D733-45.

7. Robinson JT, Thorvaldsdottir H, Winckler W, Guttman M, Lander ES, Getz G, et al. Integrative genomics viewer. Nat Biotechnol. 2011;29(1):24-6.

8. Genomes Project C, Auton A, Brooks LD, Durbin RM, Garrison EP, Kang HM, et al. A global reference for human genetic variation. Nature. 2015;526(7571):68-74.

9. Lek M, Karczewski KJ, Minikel EV, Samocha KE, Banks E, Fennell T, et al. Analysis of protein-coding genetic variation in 60,706 humans. Nature. 2016;536(7616):285-91.

10. Forbes SA, Beare D, Gunasekaran P, Leung K, Bindal N, Boutselakis H, et al. COSMIC: exploring the world's knowledge of somatic mutations in human cancer. Nucleic acids research. 2015;43(Database issue):D805-11.

11. Sherry ST, Ward MH, Kholodov M, Baker J, Phan L, Smigielski EM, et al. dbSNP: the NCBI database of genetic variation. Nucleic acids research. 2001;29(1):308-11.

12. Landrum MJ, Lee JM, Riley GR, Jang W, Rubinstein WS, Church DM, et al. ClinVar: public archive of relationships among sequence variation and human phenotype. Nucleic acids research. 2014;42(Database issue):D980-5.

13. Adzhubei I, Jordan DM, Sunyaev SR. Predicting functional effect of human missense mutations using PolyPhen-2. Current protocols in human genetics / editorial board, Jonathan L Haines [et al]. 2013;Chapter 7:Unit7 20.

14. Ng PC, Henikoff S. SIFT: Predicting amino acid changes that affect protein function. Nucleic acids research. 2003;31(13):3812-4.

15. Papaemmanuil E, Gerstung M, Malcovati L, Tauro S, Gundem G, Van Loo P, et al. Clinical and biological implications of driver mutations in myelodysplastic syndromes. Blood. 2013;122(22):3616-27; quiz 99.

16. Hou HA, Lin CC, Chou WC, Liu CY, Chen CY, Tang JL, et al. Integration of cytogenetic and molecular alterations in risk stratification of 318 patients with de novo non-M3 acute myeloid leukemia. Leukemia. 2014;28(1):50-8.

17. Hou HA, Kuo YY, Tang JL, Chou WC, Yao M, Lai YJ, et al. Clinical implications of the SETBP1 mutation in patients with primary myelodysplastic syndrome and its stability during disease progression. Am J Hematol. 2014;89(2):181-6.

18. Hou HA, Chou WC, Lin LI, Chen CY, Tang JL, Tseng MH, et al. Characterization of acute myeloid leukemia with PTPN11 mutation: the mutation is closely associated with NPM1 mutation but inversely related to FLT3/ITD. Leukemia. 2008;22(5):1075-8.

19. Lin LI, Chen CY, Lin DT, Tsay W, Tang JL, Yeh YC, et al. Characterization of CEBPA mutations in acute myeloid leukemia: most patients with CEBPA mutations have biallelic mutations and show a distinct immunophenotype of the leukemic cells. Clin Cancer Res. 2005;11(4):1372-9.

20. Tang JL, Hou HA, Chen CY, Liu CY, Chou WC, Tseng MH, et al. AML1/RUNX1 mutations in 470 adult patients with de novo acute myeloid leukemia: prognostic implication and interaction with other gene alterations. Blood. 2009;114(26):5352-61.

21. Hou HA, Liu CY, Kuo YY, Chou WC, Tsai CH, Lin CC, et al. Splicing factor mutations predict poor prognosis in patients with de novo acute myeloid leukemia. Oncotarget. 2016;7(8):9084-101.

22. Shiah HS, Kuo YY, Tang JL, Huang SY, Yao M, Tsay W, et al. Clinical and biological implications of partial tandem duplication of the MLL gene in acute myeloid leukemia without chromosomal abnormalities at 11q23. Leukemia. 2002;16(2):196-202.

23. Chen TC, Hou HA, Chou WC, Tang JL, Kuo YY, Chen CY, et al. Dynamics of ASXL1 mutation and other associated genetic alterations during disease progression in patients with primary myelodysplastic syndrome. Blood Cancer J. 2014;4:e177.

24. Lin CC, Hou HA, Chou WC, Kuo YY, Liu CY, Chen CY, et al. IDH mutations are closely associated with mutations of DNMT3A, ASXL1 and SRSF2 in patients with myelodysplastic syndromes and are stable during disease evolution. Am J Hematol. 2014;89(2):137-44.

25. Chou WC, Lei WC, Ko BS, Hou HA, Chen CY, Tang JL, et al. The prognostic impact and stability of Isocitrate dehydrogenase 2 mutation in adult patients with acute myeloid leukemia. Leukemia. 2011;25(2):246-53.

26. Chou WC, Chou SC, Liu CY, Chen CY, Hou HA, Kuo YY, et al. TET2 mutation is an unfavorable prognostic factor in acute myeloid leukemia patients with intermediate-risk cytogenetics. Blood. 2011;118(14):3803-10.

27. Falini B, Mecucci C, Tiacci E, Alcalay M, Rosati R, Pasqualucci L, et al. Cytoplasmic nucleophosmin in acute myelogenous leukemia with a normal karyotype. N Engl J Med. 2005;352(3):254-66.

28. Hou HA, Huang TC, Lin LI, Liu CY, Chen CY, Chou WC, et al. WT1 mutation in 470 adult patients with acute myeloid leukemia: stability during disease evolution and implication of its incorporation into a survival scoring system. Blood. 2010;115(25):5222-31.

29. Hou HA, Chou WC, Kuo YY, Liu CY, Lin LI, Tseng MH, et al. TP53 mutations in de novo acute myeloid leukemia patients: longitudinal follow-ups show the mutation is stable during disease evolution. Blood Cancer J. 2015;5:e331.

30. Chuang MK, Chiu YC, Chou WC, Hou HA, Chuang EY, Tien HF. A 3-microRNA scoring system for prognostication in de novo acute myeloid leukemia patients. Leukemia. 2015;29(5):1051-9.

31. Subramanian A, Tamayo P, Mootha VK, Mukherjee S, Ebert BL, Gillette MA, et al. Gene set enrichment analysis: a knowledge-based approach for interpreting genome-wide expression profiles. Proc Natl Acad Sci U S A. 2005;102(43):15545-50.

32. Cheson BD, Bennett JM, Kopecky KJ, Buchner T, Willman CL, Estey EH, et al. Revised recommendations of the International Working Group for Diagnosis, Standardization of Response Criteria, Treatment Outcomes, and Reporting Standards for Therapeutic Trials in Acute Myeloid Leukemia. J Clin Oncol. 2003;21(24):4642-9.

33. Dohner H, Estey E, Grimwade D, Amadori S, Appelbaum FR, Buchner T, et al. Diagnosis and management of AML in adults: 2017 ELN recommendations from an international expert panel. Blood. 2017;129(4):424-47.
